# A Newly Sequenced *Alcaligenes faecalis* Strain: Implications for Novel Temporal Symbiotic Relationships

**DOI:** 10.1128/genomeA.01246-14

**Published:** 2014-12-24

**Authors:** Armando Hernández-Mendoza, Luis Fernando Lozano-Aguirre Beltrán, Fernando Martínez-Ocampo, Rosa Estela Quiroz-Castañeda, Edgar Dantán-González

**Affiliations:** a Laboratorio de Investigaciones Ambientales, Centro de Investigación en Biotecnología, Universidad Autónoma del Estado de Morelos, Cuernavaca, Morelos, Mexico; b Departamento de Bioquímica y Biología Molecular, Facultad de Ciencias, Universidad Autónoma del Estado de Morelos, Cuernavaca, Morelos, Mexico; c Programa de Genómica Evolutiva, Centro de Ciencias Genómicas, Universidad Nacional Autónoma de México, Cuernavaca, Morelos, Mexico

## Abstract

We report here the draft genome sequence of *Alcaligenes faecalis* strain MOR02, a bacterium that is able to colonize nematodes in a temporary fashion and kill insects for their own benefit. The availability of the genome should enable us to explain these phenotypes.

## GENOME ANNOUNCEMENT

Associations between nematodes and bacteria have been completely described for *Heterorhabditis bacteriophora*-*Photorhabdus luminescens* and *Steinernema carpocapsae*-*Xenorhabdus nematophila* (1), but additional associations continue to be reported, which can be beneficial or harmful as well as temporary, facultative, or stably maintained. Entomopathogenic nematodes are commercially used as biological control agents for crop pests (2), and recently, Lysenko and Weiser (3) isolated bacteria associated with *S. carpocapsae*, such as *Alcaligenes* spp., *Pseudomonas* spp., and *Acinetobacter* spp., which are also pathogenic to crop pests. In this study, we report the draft genome sequence of *Alcaligenes faecalis* strain MOR02, isolated from an entomopathogenic nematode recovered from soil samples in Morelos, Mexico; the sequence shows a temporary association with *Steinernema feltiae*, *S.* carpocapsae, and *H. bacteriophora* and causes 96% mortality to *Galleria mellonella* larvae.

DNA was obtained using the AxyPrep bacterial genomic DNA miniprep kit (Axygen), and 5 µg of genomic DNA was used for whole-genome DNA sequencing using the Genome Analyzer IIx (Illumina) system. We obtained 19,250,362 paired-end reads and 315× coverage. The Illumina reads were assembled *de novo* using the SPAdes (version 3.1.1) program, with *k*-mer odd values of 21 to 71, and the SSPACE standard (version 3.0) program for scaffolding into 23 contigs. The draft genome of *A. faecalis* strain MOR02 is 4,402,705 bp, with a G+C content of 56.4%.

The 23 contigs were analyzed on the RAST (version 2.0) server (4), and 4,071 open reading frames (ORFs) were identified, including 4,019 coding sequences (CDS) and 52 RNAs. The contig1 sequence (722,086 bp) was analyzed with the Aragorn (5) and RNAmmer (version 1.2) (6) servers, and we identified genes coding for tRNA-Val(GAC) (positions 121713 to 121789), tRNA-Leu(TAG) (positions 324903 to 324987), tRNA-Met(CAT) (456223 to 456301), in addition to 5S rRNA (609890 to 610001), 23S rRNA (610185 to 613067), tRNA-Ala(TGC) (613447 to 613522), and tRNA-Ile(GAT) (613534 to 613610), as well as 16S rRNA (613709 to 615233), which was organized in a closely similar order to the reported-like operon arrangement for the *A. faecalis* subsp. *faecalis* NCIB 8687 contig (GenBank accession no. AKMR01000044; 6,924 bp). Based on the sequence of the 16S rRNA gene (1,525 bp) reported here, this strain was closest to (i) *A. faecalis* BC2000 (GenBank accession no. AY662683.1; identity, 99%), belonging to a group of bacteria isolated from rhizosphere soil that have functions on plant growth promotion and antagonism against plant parasitic nematodes; (ii) *Alcaligenes* sp. strain F78 (GenBank accession no. EU443097.1; identity, 99%), isolated from a mycorrhizosphere bacterial conglomerate; (iii) *Alcaligenes* sp. strain ECU0401 (GenBank accession no. EF535732.1), a nitrilase producer; and (iv) *Alcaligenes* sp. strain PGBS001 (GenBank accession no. EU622578.1; identity, 99%), isolated from a microbial community decomposing wheat straw under aerobic conditions. The 4,071 ORFs reported by the RAST server were compared with the Clusters of Orthologous Groups (COG), Gene Ontology (GO), KEGG Orthology (KO), and Pfam databases. The predicted proteins will be useful for predicting the genes involved in symbiosis, pathology, intracellular survival, toxicity, antibiotic production, and more, as well as to understand the phenotypic characteristics observed in this bacterium.

### Nucleotide sequence accession numbers.

This whole-genome shotgun project has been deposited at DDBJ/EMBL/GenBank under the accession no. JQCV00000000. The version described in this paper is version JQCV01000000.
